# Supplementary material for: Intramolecular and Metal-to-Molecule Charge Transfer Electronic Resonances in the Surface-Enhanced Raman Scattering of 1,4-Bis((E)-2-(pyridin-4-yl)vinyl)naphthalene
Source: Molecules. 2019 Dec 17;24(24):4622. doi: 10.3390/molecules24244622 (PMC6943491; doi:10.3390/molecules24244622)
Supplement: Supplementary file 1 [file molecules-24-04622-s001.pdf]

molecules

Supplementary Materials

Intramolecular and metal-to-molecule charge transfer electronic resonances in the surface-enhanced Raman scattering of 1,4-Bis((E)-2-(pyridin-4-yl)vinyl)naphthalene

Isabel López-Tocón[1]*, Elizabeth Imbarack[2], Juan Soto[1], Santiago Sanchez-Cortes[3], Patricio Leyton[2] and Juan Carlos Otero[1]*

[1] Andalucía Tech, Unidad Asociada IEM-CSIC, Departamento de Química Física, Facultad de Ciencias, Universidad de Málaga, Málaga, Spain; tocon@uma.es , soto@uma.es, jc_otero@uma.es

[2] Instituto de Química, Pontificia Universidad Católica de Valparaiso, Valparaiso, Chile; eimbarack@yahoo.es, patricio.leyton@pucv.cl

[3] Instituto de Estructura de la Materia, Consejo Superior de Investigaciones Científicas, Madrid, Spain; s.sanchez.cortes@csic.es

* Correspondence: tocon@uma.es; jc_otero@uma.es

Received: date; Accepted: date; Published:

Table S1. Assignment of the main lines recorded in the Raman and SERS spectra and the experimental and CAM-B3LYP/def2-TZVPP calculated wavenumbers (cm^{-1}) of bpyvn and of the Ag_2-bpyvn complex.

Experimental[1]	Calculated bpyvn	Calculated Ag_2-bpyvn	Assignment[2]
1625(vs)	1733	1733	ν(C=C)
1592(m)	1675	1675	8a;ν(CC)py
1575(vs)	1658	1659	8a;ν(CC)naph
1368(s)	1405	1405	δ(CH)naph
1337(s)	1391	1393	14;δ(CC)py
1202(s)	1260	1263	9a;δ(CH)py
988(m)	1031	1031	12;δring;py
969(m)	1022	1023	γ(CH)py,naph

[1]vs: very strong, s: strong, m: medium. [2]py: pyridine, naph: naphthalene, ν: stretching, δ: In-plane deformation, γ: out-of-plane deformation. Wilson's nomenclature from Varsanyi, G. Vibrational spectra of benzene derivatives, Academic Press, New York, 1969.

Table S2. SERS wavenumbers (cm^{-1}) of the strongest bands of bpyvn at different electrode potentials (785 nm excitation).

**Experimental**	**Electrode Potential/V**											
**Raman[1]**	**OC[2]**	**0.0**	**-0.1**	**-0.2**	**-0.3**	**-0.4**	**-0.5**	**-0.6**	**-0.7**	**-0.8**	**-0.9**	**-1.0**
1625(vs)	1624	1626	1626	1626	1625	1626	1626	1626	1626	1626	1625	1625
1592(m)	1606	1607	1607	1607	1607	1606	1606	1605	1604	1604	1603	1603
1575(vs)	1572	1573	1574	1574	1574	1573	1574	1573	1573	1573	1572	1572
1368(s)	1368	1367	1368	1368	1367	1367	1367	1367	1367	1368	1368	1368
1337(s)	1337	1338	1336	1336	1336	1336	1336	1335	1336	1337	1337	1337
1202(s)	1206	1206	1207	1206	1206	1205	1205	1204	1204	1204	1203	1203
988(m)	1012	1013	1011	1011	1011	1011	1010	1008	1008	1009	1010	1010
969(m)	967	968	966	966	966	966	966	966	966	966	966	966

[1]vs: very strong, s: strong, m: medium. Raman spectrum of the solid. [2]SERS recorded at open-circuit (OC).

Table S3. SERS wavenumbers (cm^{-1}) of the strongest bands of bpyvn at different electrode potentials (514.5 nm excitation).

**Experimental**	**Electrode Potential/V**											
**Raman[1]**	**OC[2]**	**0.0**	**-0.1**	**-0.2**	**-0.3**	**-0.4**	**-0.5**	**-0.6**	**-0.7**	**-0.8**	**-0.9**	**-1.0**
1625(vs)	1626	1626	1626	1626	1626	1626	1626	1627	1627	1627	1627	1627
1592(m)	1606	1606	1606	1606	1606	1606	1606	1605	1606	1605	1605	1605
1575(vs)	1575	1573	1573	1574	1574	1574	1574	1574	1574	1574	1574	1574
1368(s)	1367	1367	1367	1367	1367	1367	1367	1367	1367	1367	1367	1367
1337(s)	1337	1337	1336	1337	1336	1337	1335	1336	1335	1335	1335	1335
1202(s)	1207	1207	1206	1206	1206	1206	1206	1206	1207	1208	1208	1208
988(m)	1010	1009	1010	1010	1010	1010	1010	1010	1010	1008	1008	1008
969(m)	969	967	966	964	965	966	966	966	966	961	961	962

[1]vs: very strong, s: strong, m: medium. Raman spectrum of the solid. [2]SERS recorded at open-circuit (OC).

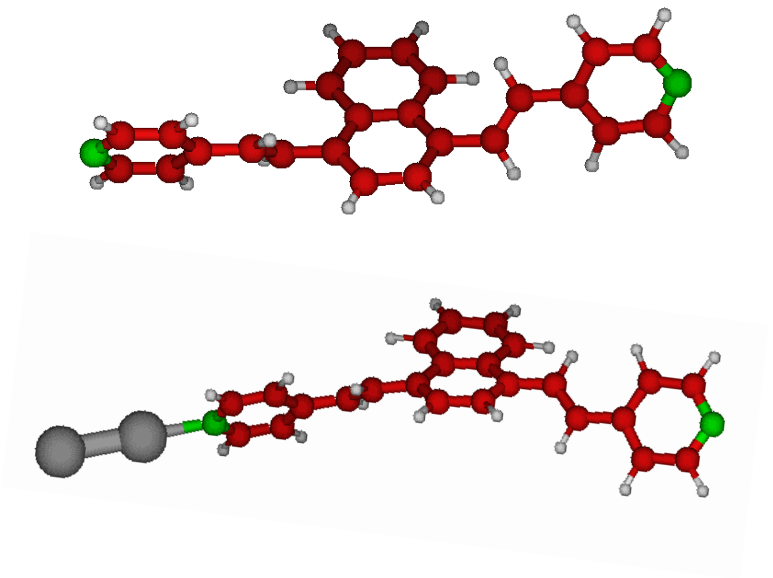

Figure S1. CAM-B3LYP/def2-TZVPP optimized structure of the bpyvn (top) and of the Ag_2-bpyvn system modeling the surface complex (bottom).

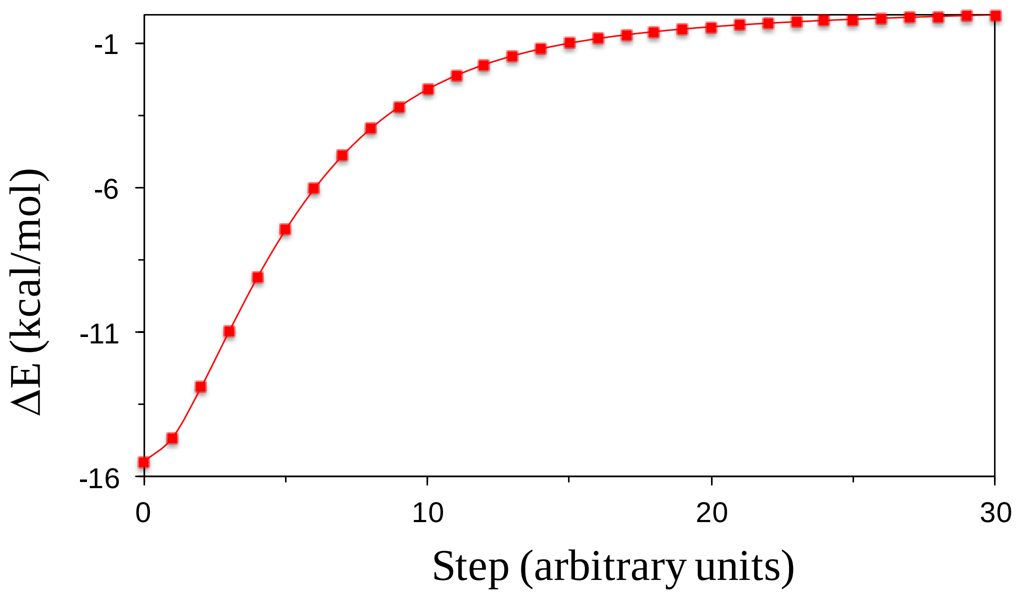

Figure S2. CAM-B3LYP/def2-TZVPP potential energy profile (interpolation line) for association/dissociation of bpyvn to the Ag_2 cluster. Step 0: d(N-Ag)=2.263 Å; Step 30: d(N-Ag)=6.700 Å.

Computational resonance Raman spectra

The modified mathematic expression of the intensity I_k of a Raman band of wavenumber k under preresonance conditions given initially by Long [1] becomes:

$$I_k = \frac{(2\pi)^4}{h^2} K N_{0^i} (\tilde{\nu}_1 - \tilde{\nu}_k)^4 \tilde{\nu}_k{}^2 \sum_e (W^e_{\Gamma_{k,e}} \Delta_{k,e})^2 \quad (1)$$

where the integrals of vibrational overlapping, $\Delta_{k,e}$, are obtained using the recurrence formulae of Manneback [2] with the gradient approximation:

$$\Delta_{k,e} = \frac{1}{\sqrt{2}} 2.408 \cdot 10^6 \, \tilde{\nu}^{-3/2} f_e M^{-1/2} L_K \quad (2)$$

being, f_e the force row vector of electronic state *e*, *M* is the 3Nx3N diagonal matrix of atomic masses, and L_K is the column eigenvector of the Hessian matrix for mode *k*-th.

Other parameters given in equation (1) are: K is a constant for a given experimental condition and a given irradiance of the incident radiation of wavenumber $\tilde{\nu}_1$; N_{0^i} is the Boltzmann population of the vibrational ground state; $\tilde{\nu}_k$ is the wavenumber of *k*-th mode; $W^e_{\Gamma_{k,e}}$ is given by equation:

$$W^e_{\Gamma_{k,e}} = \frac{|\mu_e^0|^2}{\aleph \left[(\tilde{\nu}_e - \tilde{\nu}_1)^2 + \Gamma^2_{k,e}\right]^{1/2} \left[(\tilde{\nu}_e + \tilde{\nu}_k - \tilde{\nu}_1)^2 + \Gamma^2_{k,e}\right]^{1/2}} \quad (3)$$

and depends on the transition dipole moment, $\mu_e{}^0$; the energy difference between the excited electronic state *e* and the ground state, $\tilde{\nu}_e$; the wavenumber $\tilde{\nu}_k$ of *k* mode and the damping factor, $\Gamma_{k,e}$, which is related to the lifetime of the transition and is taken as 0.2 $\tilde{\nu}_k$ in this work. ℵ is a normalization constant given by:

$$\aleph = \left[\sum_e \frac{|\mu_e^0|^4}{\left[(\tilde{\nu}_e - \tilde{\nu}_1)^2 + \Gamma_{k,e}\right] \left[(\tilde{\nu}_e + \tilde{\nu}_k - \tilde{\nu}_1)^2 + \Gamma^2_{k,e}\right]} \right] \quad (4)$$

References

1. Long, D.A. in the Raman effect: A unified treatment of the theory of Raman Scattering by molecules, John Wiley&Sons, Ltd. West Sussex England, 2002.
2. Manneback, C. Computation of the intensities of vibrational spectra of electronic bands in diatomic molecules. *Physica* **1951**, *17*, 1001–1010.

© 2019 by the authors. Submitted for possible open access publication under the terms and conditions of the Creative Commons Attribution (CC BY) license (http://creativecommons.org/licenses/by/4.0/).
